# Cardiac Involvement in Cryoglobulinemia: Clinical Characteristics, Radiological Features, and Outcomes

**DOI:** 10.3390/jcm15135262

**Published:** 2026-07-06

**Authors:** Hongxiao Han, Kaini Shen, Yubo Guo, Lu Zhang, Yining Wang, Zhuang Tian, Jian Li

**Affiliations:** 1 Department of Internal Medicine, Peking Union Medical College Hospital, Chinese Academy of Medical Sciences and Peking Union Medical College, Beijing 100730, China; hanhongxiao@pumch.cn; 2 Department of Hematology, Peking Union Medical College Hospital, Chinese Academy of Medical Sciences and Peking Union Medical College, Beijing 100730, China; 3 Department of Radiology, Peking Union Medical College Hospital, Chinese Academy of Medical Sciences and Peking Union Medical College, Beijing 100730, China; 4 Department of Cardiology, Peking Union Medical College Hospital, Chinese Academy of Medical Sciences and Peking Union Medical College, Beijing 100730, China; 5 Center for Rare Diseases, State Key Laboratory of Complex Severe and Rare Diseases, Chinese Academy of Medical Sciences and Peking Union Medical College, Beijing 100730, China

**Keywords:** cryoglobulinemia, cardiac involvement, heart failure, rituximab, bortezomib

## Abstract

**Background**: Cardiac involvement in cryoglobulinemia (CG) is rare but potentially fatal, and its clinical spectrum remains poorly characterized. **Methods**: This retrospective study enrolled 11 patients with cardiac involvement among 885 patients with CG at Peking Union Medical College Hospital between January 2015 and March 2026. We analyzed its clinical characteristics, radiological features and management. **Results**: Among 885 CG patients, 11 (1.2%; 4 type I, 7 type II) had cardiac involvement. Cardiac symptoms included dyspnea (*n* = 6), chest tightness (*n* = 4), edema (*n* = 3), and orthopnea (*n* = 1). All patients had elevated N-terminal pro-B-type natriuretic peptide (median 29,799 pg/mL). Echocardiography, performed in all 11 patients, revealed left heart enlargement (*n* = 9), reduced left ventricular ejection fraction (*n* = 7), myocardial disease (*n* = 6), pericardial effusion (*n* = 4), and pulmonary hypertension (*n* = 3). Cardiac magnetic resonance in 5 of 11 patients showed non-ischemic late gadolinium enhancement in two cases. For first-line therapy, 6 of 11 patients received rituximab-based regimens, 3 of 11 received bortezomib-based regimens, and 1 of 11 received antiviral therapy with corticosteroids; 1 patient declined treatment. All 10 treated patients achieved initial cardiac improvement, with 5 relapsing and 2 dying during a median follow-up of 57 months (range 9–130 months). The estimated 4-year overall and progression-free survival rates were 77.9% (95% CI: 0.546–1.000) and 50.0% (95% CI: 0.269–0.929), respectively. **Conclusions**: Cardiac involvement in CG is rare and associated with diverse structural and functional abnormalities. Cardiac involvement should be considered in CG patients presenting with unexplained cardiac manifestations after excluding alternative causes. B-cell-targeted therapy induced an initial response, but relapse is common. Early intervention is essential given the substantial relapse burden and potential for severe morbidity.

## 1. Introduction

Cryoglobulinemia (CG) is a condition characterized by the presence of circulating cryoglobulins, which are immunoglobulins that reversibly precipitate at 4 °C and redissolve upon rewarming to 37 °C. They are categorized into three distinct types based on their immunoglobulin constituents [1]: Type I, comprising monoclonal immunoglobulin, demonstrates an association with hematologic malignancies, encompassing monoclonal gammopathy of clinical significance (MGCS), Waldenström’s macroglobulinemia, B-cell non-Hodgkin lymphoma (B-NHL), and multiple myeloma [2,3]. Type II features monoclonal immunoglobulin-exhibiting rheumatoid factor (RF) activity capable of binding polyclonal immunoglobulin, while Type III (polyclonal immunoglobulin) is often secondary to hepatitis C virus (HCV) and viral infections attributable to HCV and hepatitis B virus (HBV), in addition to various connective tissue disorders [4]. CG clinical symptomatology extends across a broad continuum from completely asymptomatic presentations to potentially fatal adverse events, exemplified by acute cardiac failure. Cutaneous tissue is the most commonly involved organ, with renal, musculoskeletal, and peripheral nervous systems subsequently affected [4,5]. Although involvement of vital organs (central nervous system, gastrointestinal tract, myocardium, and lungs) is uncommon, it is associated with substantial morbidity and mortality [5,6].

To date, due to the scarcity of cardiac involvement in CG, limited studies and case series have been reported. Cardiac manifestations occur in <5% of CG patients [7]. Remarkably, even without established cardiovascular risk factors, subjects demonstrating cardiac involvement retain susceptibility toward developing acute myocardial infarction [6,8]. There are also reports of pericarditis, severe heart failure, or cardiomyopathy responsible for CG complications [5]. As an immune-complex-mediated vasculitis, CG can systematically affect the heart [9]. However, the precise mechanisms remain incompletely understood. Postmortem evidence suggests that cryoglobulins may cause myocardial injury via microvascular vasculitis, typically sparing the epicardial coronary arteries [10,11,12]. This pattern supports the hypothesis that heart failure in CG results primarily from small-vessel necrotizing vasculitis rather than large-artery disease. Notably, most affected patients have no prior cardiac history, implicating vasculitis as the likely primary cause, although definitive histopathological confirmation is still lacking [13].

Despite the low reported frequency, cardiac involvement warrants a dedicated investigation because it can lead to rapidly progressive heart failure and sudden cardiac death, and its management differs from that of primary cardiac diseases. However, existing studies lack systematic descriptions of the cardiac involvement spectrum, including detailed clinical phenotypes, imaging characteristics, treatment responses, and long-term outcomes.

To address these gaps, we studied the largest cohort of CG patients with cardiac involvement to date, with the longest follow-up period as yet reported. Our detailed analysis of clinical presentations, imaging features, treatment approaches, and long-term outcomes is intended to help clinicians recognize cardiac involvement early and guide therapeutic choices. Through this work we offer a useful framework for improving the diagnosis and management of this rare but life-threatening condition.

## 2. Materials and Methods

### 2.1. Patients

For this retrospective analysis, we recruited CG patients receiving treatment at Peking Union Medical College Hospital during the period from January 2015 to March 2026. serum cryoglobulin positivity. The inclusion criteria were serum cryoglobulin positivity and cardiac involvement defined as follows: in patients with confirmed CG, after systematic exclusion of alternative etiologies, the diagnosis required both ≥1 cardiac clinical manifestation and ≥1 objective cardiac abnormality on at least one of the following investigations: reduced left ventricular ejection fraction (LVEF); left ventricular (LV) and/or left atrial (LA) enlargement; myocardial disease; non-ischemic late gadolinium enhancement (LGE) on cardiac magnetic resonance (CMR); pulmonary hypertension; pericardial effusion; elevated serum N-terminal pro-B-type natriuretic peptide (NT-proBNP) or B-type natriuretic peptide (BNP) and/or elevated cardiac troponin I (cTnI); and perivascular inflammation on biopsy. Treatment response was supportive but not required for diagnosis. Systematic exclusion of alternative etiologies included coronary artery disease (no ischemic changes on electrocardiography, echocardiography, or CMR; hypertensive heart disease (no hypertension or significant LV hypertrophy); viral myocarditis (no preceding viral prodrome); infiltrative cardiomyopathy (no restrictive pattern on echocardiography or CMR); renal volume overload (no cardiac improvement after volume reduction); pulmonary hypertension (no secondary causes and improved with CG-directed therapy); treatment-related cardiotoxicity (no exposure to cardiotoxic agents). The requirement for written informed consent was waived by the ethics committee due to the retrospective design and use of anonymized clinical data, and the Institutional Review Board of Peking Union Medical College Hospital evaluated and sanctioned the research protocol (approval number I-26PJ0297). This study adhered to the ethical principles stipulated in the 1964 Declaration of Helsinki along with subsequent revisions.

### 2.2. Clinical, Laboratory, and Radiologic Imaging Data

Clinical information was gathered, encompassing demographic features, clinical symptoms, concurrent medical comorbidities, laboratory examinations, therapeutic interventions, and survival outcomes. CG-related clinical manifestations corresponded to those documented in an earlier investigation [14]. Cryoglobulin detection was performed according to established methodologies described in prior studies [15,16], while cardiac evaluation was performed using electrocardiography, echocardiography, and CMR (utilizing a 3.0 Tesla scanner, MAGNETOM Skyra, Siemens Healthcare, Erlangen, Germany).

### 2.3. Definition of Underlying Diseases

The predisposing conditions comprised the following: (I) Hematological disorders: MGCS and B-NHL diagnoses were established in accordance with current internationally recognized diagnostic criteria [17,18]. (II) Infectious etiologies: Encompassing the following: (a) HCV viral infection, demonstrated by seropositivity for anti-HCV antibodies together with HCV-RNA detectable in serum by polymerase chain reaction (PCR) assay; (b) HBV viral infection, evidenced by seropositivity for HBV surface antigen (HBsAg) within serum, and/or measurable HBV-DNA in serum. (III) Primary Sjögren’s syndrome (pSS): Assessed according to the 2016 classification criteria established jointly by the American College of Rheumatology (ACR) and the European League Against Rheumatism (EULAR) [19].

### 2.4. Treatment and Outcomes

Therapeutic interventions were categorized into four distinct groups according to the primary therapeutic agent: (I) rituximab-containing regimens; (II) bortezomib-containing regimens; (III) combined rituximab–bortezomib protocols; (IV) antiviral agents administered concurrently with corticosteroids. Cardiac response was assessed based on clinical follow-up, echocardiography (LVEF, chamber dimensions, pericardial effusion, pulmonary artery systolic pressure), and cardiac biomarkers (NT-proBNP, cTnI). Cardiac status was classified as improved (improvement of symptoms, cardiac functional parameters on echocardiography, and a decrease in NT-proBNP/cTnI by >50% from baseline), stable (no significant change), or worsened (worsening of symptoms, cardiac functional parameters, and an increase in cardiac biomarkers). In patients who did not undergo follow-up imaging, we relied mainly on clinical symptoms and biomarker trends for evaluation. Relapse was defined as clinical signs of vasculitis reappearing in any organ after remission, with or without a renewed cryoglobulin concentration elevation after a previous decrease or negative value. Isolated cryoglobulin concentration evaluation without clinical signs was not considered relapse. Other organ involvement was assessed as a secondary outcome and qualitatively classified as improved, stable, or worsened based on medical records.

Overall survival (OS) was computed from initial diagnosis until a mortality event or final follow-up assessment. Progression-free survival (PFS) was defined as the time from the initial CG-directed therapy date to the first occurrence of any of the following: relapse, death from any cause, or last follow-up. Patients who were lost to follow-up were censored at the date of the last known living status confirmation. The last follow-up was in March 2026.

### 2.5. Statistical Analysis

Descriptive statistics were applied to summarize the clinical and imaging data. Continuous variables were presented as medians with ranges, and categorical variables as frequencies with percentages. Survival outcomes were analyzed using the Kaplan–Meier method, and survival curves were compared using the log-rank test where appropriate. Missing data were not imputed; all analyses were based on available data. The evaluable patient number for each variable was indicated where applicable. All statistical analyses were performed using R (version 4.5.2) and a two-sided *p*-value < 0.05 was considered statistically significant.

## 3. Results

### 3.1. Patient Characteristics

Overall, 885 individuals were identified, comprising 172 subjects (19.4%) presenting with type I CG, 141 (15.9%) with type II CG, and 572 (64.6%) with type III CG, all of whom received a CG diagnosis at Peking Union Medical College Hospital during the interval from January 2015 through to March 2026. Collectively, eleven (1.2%) patients fulfilled the inclusion criteria, consisting of four type I patients and seven type II patients of CG. Among these 11 subjects exhibiting cardiac involvement, the cohort comprised 5 males and 6 females, with a median age at time of diagnosis of 53 years (spanning 26 to 71 years) and a median time interval from initial symptom manifestation to disease confirmation of 8 months (extending from 1 to 76 months). Baseline demographic, clinical, and laboratory characteristics of the 11 patients are summarized in Table 1.

Every subject with type I CG had secondary causes attributable to hematological disorders, including MGCS (3 of 11, 27.3%) and B-NHL (1 of 11, 9.1%). Type II CG was identified in seven patients: two with HCV infection (18.2%), one with HBV infection (9.1%), one with B-NHL (9.1%), one with pSS (9.1%), and two with no identifiable secondary etiology (18.2%). The median number of affected organs reached four (spanning from two to six). Beyond cardiac involvement, the organs most frequently affected by CG were skin (8 of 11, 72.7%) and kidney (8 of 11, 72.7%), followed by the peripheral nerves (5 of 11, 45.4%), joint (4 of 11, 36.4%), lung (2 of 11, 18.2%), and gastrointestinal tract (GI) (1 of 11, 9.1%).

Every subject exhibiting cardiac involvement manifested sustained cardiac symptomatology, encompassing dyspnea (6 of 11, 54.5%), chest tightness (4 of 11, 36.4%) edema (3 of 11, 27.3%) and orthopnea (1 of 11, 9.1%) Additional predominant clinical presentations comprised proteinuria (8 of 11, 72.7%)alongside renal function impairment (6 of 11, 54.5%) which were subsequently followed by cutaneous purpura (5 of 11, 45.5%), gross hematuria (4 of 11, 36.4%), sensory polyneuropathy (4 of 11, 36.4%), articular arthralgia (3 of 11, 27.3%), and livedo reticularis (2 of 11, 18.2%), together with mucocutaneous ulceration (2 of 11, 18.2%). Additionally, one patient (#9) exhibiting GI involvement manifested abdominal pain, diarrhea, and bloody watery stools with tenesmus, and the two patients (#3, #5) with pulmonary involvement presented with diffuse alveolar hemorrhage (DAH) and interstitial lung disease, respectively, which we have described previously [20].

### 3.2. Laboratory Findings

Of all patients with cardiac involvement, three (#7, #8, and #9) of ten evaluable patients had an increase in cTnI value, with a median concentration of 0.454 μg/L (range of 0.120–3.657 μg/L) among these 3 patients. One patient (#11) had an increase in high-sensitivity cTnI, reaching a value of 54 ng/L. Likewise, NT-proBNP was elevated in all 11 patients (median of 29,799 pg/mL; range of 209–>35,000 pg/mL), and BNP was elevated in all seven tested patients (median of 340 ng/L; range of 108–>5000 ng/L). These results are detailed in Table 2.

Among patients with cardiac involvement, the median serum cryoglobulin concentration was 856.7 mg/L (range of 62.6–4576.7 mg/L), and seven had an IgM κ; three had an IgG, including two IgG λ and one IgG κ; and one had an IgA κ isotype. Furthermore, serum immunofixation electrophoresis (IFE) was positive in seven patients overall, while urinary IFE demonstrated positivity in four out of the ten assessable subjects. Notably, across all 11 patients, the isotypes of monoclonal immunoglobulin corresponded to the isotypes of cryoglobulins.

RF was elevated in 8 of 11 patients (72.7%), while in the overall cohort, the median RF concentration was 253.8 IU/mL (range of 3.0–617.7 IU/mL). Serum C3 and C4 were measured in all patients: reduced C3 was observed in four patients (36.4%), with a median C3 level of 0.777 g/L (range of 0.283–1.435 g/L) across all patients; reduced C4 was found in seven patients (63.6%), with a median C4 level of 0.048 g/L (range of 0.001–0.217 g/L).

### 3.3. Cardiac Investigations

Cardiac findings, including electrocardiography, echocardiography, CMR, and histopathology, are detailed in Table 2, with representative images shown in Supplementary Figure S1. Electrocardiography was available in all 11 patients. Abnormalities were observed in seven cases (63.6%), including sinus tachycardia (4 of 11, 36.4%), low and flat T-waves (4 of 11, 36.4%), and T-wave inversion in precordial leads (1 of 11, 9.1%); the remaining five patients had normal electrocardiography. All 11 patients underwent echocardiography, with the following results: nine (81.8%) had enlarged cardiac chambers, including LV and/or LA enlargement; seven patients (63.6%) had reduced LVEF; six (54.5%) had echocardiographic evidence of myocardial disease; four (36.4%) had pericardial effusion; and three (27.3%) had pulmonary hypertension. Among them, Patient #9 exhibited a distinctive non-ischemic wall motion pattern characterized by basal segmental preservation with apical septal akinesia and global hypokinesis, which is not consistent with coronary artery distribution. Detailed echocardiographic parameters are summarized in Table 3. Owing to contraindications, limited local CMR availability, and the retrospective data collection design, CMR was performed in five patients, and corroborated echocardiographic findings while providing enhanced tissue characterization. Among the five patients who underwent CMR, two showed non-ischemic LGE: Patient #1 had subepicardial LGE in the LV lateral and inferior walls, and Patient #11 had mid-myocardial LGE in the LV free wall. No patient exhibited subendocardial or transmural LGE suggestive of coronary artery disease. In addition, endomyocardial biopsy in Patient #4 demonstrated moderate perivascular mononuclear cell infiltration.

### 3.4. Treatment and Outcomes

Comprehensive therapeutic interventions and clinical outcomes for all CG subjects exhibiting cardiac involvement are detailed in Table 4. One patient (#1) declined treatment and was lost to follow-up, while six (54.5%) were administered rituximab-containing protocols as initial therapy, encompassing rituximab combined with corticosteroids (2 of 11, 18.2%), DRC (rituximab, cyclophosphamide, and dexamethasone) (2 of 11, 18.2%), DRC plus antiviral therapy for HCV infection (1 of 11, 9.1%), and R-CVP (rituximab, cyclophosphamide, vincristine, together with prednisone) plus antiviral therapy for HCV infection (1 of 11, 9.1%). Three (27.3%) patients were administered bortezomib-containing regimens as initial therapy, including BCD (bortezomib, cyclophosphamide, and dexamethasone) in two patients (18.2%) and BCD with plasma exchange in one patient (9.1%). Patient #6 received antiviral therapy for HBV infection and corticosteroids as first-line treatment.

All ten treated patients showed cardiac improvement: in all cases, symptoms improved and cardiac biomarkers (NT-proBNP or cTnI) normalized or decreased, and among the seven patients with follow-up echocardiography, all demonstrated improved LVEF, reduced chamber diameters, decreased pericardial effusion and decreased pulmonary artery systolic pressure (Table 3). The remaining three patients lacked post-treatment imaging but showed clinical and biomarker improvement. Other organ involvement improved in all ten patients during the early post-treatment period.

Overall, among the ten treated patients, five achieved sustained remission; five experienced relapse. Among these five patients, Patient #3 died of progressive B-NHL, and the remaining four received second-line therapy with Rd (lenalidomide plus dexamethasone), corticosteroids plus antiviral therapy for HBV infection, DRC, or rituximab monotherapy (Table 5). Of these, three achieved remission. Patient #7 died of respiratory failure. It should be noted that Patient #11 had isolated skin relapse with stable cardiac status.

**Figure 1:**
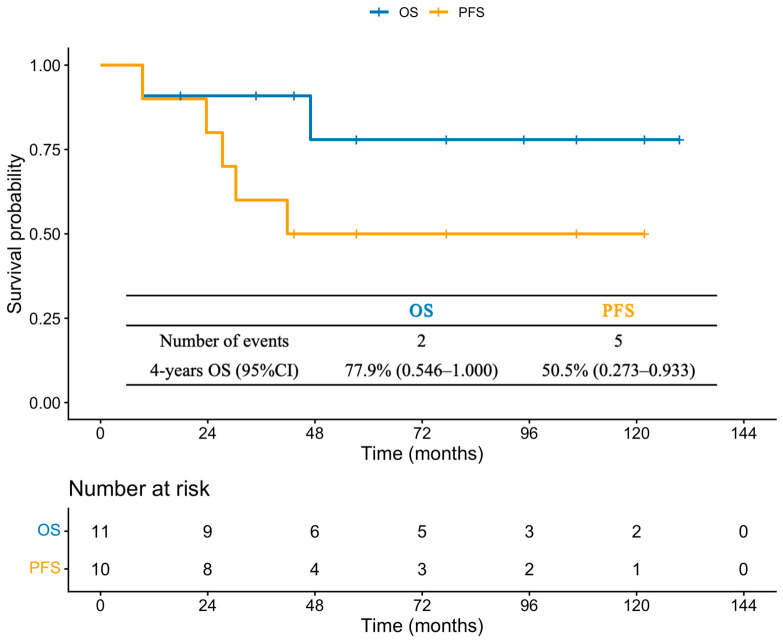
Overall survival (OS) and progression-free survival (PFS) of the whole cohort (*n* = 11). Patient #1 (declined treatment and lost to follow-up) was censored at the date of last known living status confirmation.

Among the eleven patients, the median follow-up duration was 57 months (range of 9–130 months), and the estimated 4-year OS and PFS rates for the entire cohort were 77.9% (95% CI: 0.546–1.000) and 50.0% (95% CI: 0.269–0.929), respectively (Figure 1). 

## 4. Discussion

Cardiac involvement in CG remains infrequently reported. This investigation comprising 11 subjects represents one of the most extensive documented series of CG subjects exhibiting cardiac involvement to date. For the first time, we have systematically characterized the clinical profiles, imaging findings, and therapeutic outcomes of CG accompanied by cardiac involvement in a well-defined cohort.

In the present study, cardiac involvement occurred in 1.2% of CG cases. This occurrence rate was reduced compared with the 4.2% documented by Terrier et al. [13] in HCV-related CG, yet aligned closely with the 1.2% observed by Ghembaza et al. [21] among type I CG patients. Notably, another retrospective study of 231 mixed CG patients identified no cardiac involvement [22]. These findings demonstrate that cardiac involvement in CG is uncommon, and population-based incidence data remained unavailable at that time. Cardiac involvement exclusively manifested among four type I and seven type II CG patients, with no cases observed among type III CG in this cohort. A previous study found that cardiac manifestations were independently associated with B-NHL and GI involvement by multivariate analysis of 165 patients with HCV-related mixed CG [13]. Notably, in our cohort, two patients had B-NHL and one had GI involvement, which is consistent with these findings. However, given the small sample size, no definitive conclusions can be drawn regarding subtype-specific associations.

Cardiac symptoms manifested diversely in our cohort, consisting of dyspnea (6 of 11, 54.5%), chest tightness (4 of 11, 36.4%) edema (3 of 11, 27.3%) and orthopnea (1 of 11, 9.1%). In addition to the heart, we found that the skin and kidneys represented the most frequently affected organs, similar to the findings of prior studies documenting the most frequently affected organ systems in CG [4,22,23]. Furthermore, we identified potentially fatal manifestations, encompassing DAH affecting Subject #3 together with the GI tract affecting Patient #9.

An earlier investigation of 54 HCV-associated CG subjects demonstrated markedly elevated serum NT-proBNP concentrations relative to control subjects, implying that its increase may indicate CG-associated subclinical cardiac dysfunction [24]. Likewise, we found that all assessable subjects exhibited a significant increase in NT-proBNP (median of 29,799 pg/mL) or BNP, which supported cardiac involvement of CG patients. Hence, cardiac involvement should be considered among CG patients with an increase in NT-proBNP. However, it should be noted that NT-proBNP lacks specificity in patients with renal impairment; therefore, elevated levels should be interpreted in conjunction with other clinical and imaging findings.

Imaging characterization of cardiac involvement in CG remains scarce. Baseline electrocardiography and echocardiography in our cohort revealed a high prevalence of cardiac abnormalities, including left heart enlargement (9/11, 81.8%), reduced LVEF (7/11, 63.6%), myocardial disease (6/11, 54.5%), pericardial effusion (4/11, 36.4%), and pulmonary hypertension (3/11, 27.3%). These findings confirm that structural and functional cardiac involvement is common in this population. Of particular interest is patient #9, who exhibited a distinctive non-ischemic wall motion pattern characterized by apical septal akinesia with basal segmental preservation and global hypokinesia. This distribution does not conform to any single coronary artery territory, arguing against an atherosclerotic ischemic origin. Instead, it points toward microvascular injury or inflammatory myocardial damage of CG, consistent with prior reports [10,11,12].

Terrier et al. [13] reported myocardial perfusion defects on scintigraphy in two patients and LGE (diffuse nodular enhancement in two and subendocardial enhancement in one) on CMR in three others, implicating myocardial injury as a potential cardiac involvement mechanism. In our cohort, five patients underwent CMR. Two patients demonstrated non-ischemic LGE: subepicardial in the LV lateral and inferior walls, and mid-myocardial in the LV free wall. These patterns are most consistent with myocarditis-like injury and microvascular disease. No patient exhibited subendocardial or transmural LGE following a coronary artery distribution, which does not support ischemic injury as the primary mechanism. Infiltrative disease is also unlikely given the absence of diffuse subendocardial LGE or restrictive physiology. While the remaining three patients showed no LGE, possibly reflecting isolated microvascular dysfunction [10,13].

A recent study on diagnostic performance of multimodality imaging in cardiac masses demonstrated that CMR offers the highest single-modality diagnostic accuracy (AUC 0.94), and its combination with ^18^F-FDG PET achieves excellent performance (AUC 0.97), while echocardiography remains the widely recognized first-line imaging tool for cardiac evaluation [25]. Although that study addressed a different disease spectrum, its methodological insight, that combined imaging modalities outperform any single test, supports our multimodality approach. In our cohort, the available electrocardiographic, echocardiographic, and CMR data, when interpreted together, provided complementary information on cardiac structure, function, and tissue characteristics. The non-ischemic LGE patterns pointed toward myocarditis-like injury rather than ischemic cardiomyopathy or amyloidosis, while the absence of LGE in others did not exclude cardiac involvement, as isolated microvascular dysfunction may be the predominant mechanism. Importantly, the combined use of these modalities strengthened the diagnostic confidence for cardiac involvement beyond what any single test could provide. Future studies should consider a structured multimodality approach when feasible.

We acknowledge that our diagnostic criteria for cardiac involvement are broad and lack histopathological confirmation in all but one patient. Endomyocardial biopsy was performed only in one patient due to its invasive nature and associated risks. Although supportive laboratory findings, imaging findings, and treatment response suggest causality, caution is warranted. Future studies should combine systematic biopsy with advanced imaging and biomarkers to improve diagnostic accuracy.

Therapeutic strategies must be individualized according to the underlying etiology together with the organ impairment severity. Standardized management guidelines remain challenging to establish owing to the marked heterogeneity regarding clinical manifestations alongside underlying pathophysiological mechanisms [26]. The Italian Study Group on Cryoglobulinemia (GISC) advocated for rituximab as an efficacious and secure therapeutic alternative for severe, non-fatal manifestations, and it has demonstrated superior efficacy over conventional immunosuppressive therapies [27,28]. In our cohort of patients with B-cell non-Hodgkin lymphoma, rituximab-based chemotherapy was prioritized to target the underlying lymphoproliferative disorder. In patients with MGCS, bortezomib-based regimens were administered, aiming at the plasma cell clone. For patients with HCV or HBV infection, antiviral therapy served as the cornerstone of treatment, with or without additional immunosuppressive agents. Of note, one patient with rapidly progressive glomerulonephritis, a life-threatening cryoglobulinemic manifestation, received BCD combined with PE, reflecting the need for more intensive intervention in severe cases. For patients without an identifiable underlying etiology, the presence of severe cardiac or other vital organ involvement justified prompt and active treatment, with rituximab-based regimens being preferentially considered given their established efficacy and favorable safety profile [26].

In our cohort, initial regimens included rituximab-based protocols in six patients, bortezomib-based protocols in three, and antiviral therapy with corticosteroids in one. All treated patients showed cardiac improvement, although the degree of response varied. While B-cell depletion strategies were associated with clinical improvement in this small cohort, these observations remain descriptive and should be interpreted with caution given the limited sample size and heterogeneous treatment regimens. However, the presence of irreversible heart damage and life-threatening manifestations in CG patients with cardiac involvement correlated with poor prognosis relative to those lacking cardiac involvement [4,21]. Considering the elevated recurrence rate, patients with cardiac CG required implementation of a complete therapeutic course during initial treatment, coupled with vigilant monitoring during remission to detect early signs of recurrence and promptly initiate appropriate interventions.

This study has several strengths, most notably representing the largest and longest follow-up cohort of cardiac involvement in cryoglobulinemia to date, thereby providing important reference data for this patient population. However, several limitations should be acknowledged. The single-center retrospective design, conducted at a national referral center for rare diseases, may have enriched the cohort with more severe cases and limited the generalizability of our findings. In addition, the small sample size (*n* = 11) and the lack of histopathological confirmation by endomyocardial biopsy (performed in only one patient) further constrain the interpretation of our results. Despite these limitations, in this study we provide the largest systematic characterization of cardiac involvement in CG to date, addressing a critical knowledge gap.

## 5. Conclusions

Cardiac involvement was extremely rare in CG, which manifested diversely, encompassing diminished left ventricular systolic performance and myocardial disease, together with left heart enlargement, and often suggested therapeutic challenges and a high recurrence rate. We should consider cardiac involvement in CG patients presenting with unexplained cardiac manifestations after excluding alternative causes. While B-cell-targeted therapies (rituximab and bortezomib) achieved initial remission, relapses occurred frequently.

## Figures and Tables

**Table 1:** Demographic, clinical, and laboratory characteristics of patients with cardiac involvement.

Patient	Sex, Age	Underlying Disease	Other Involved Organ	Cardiac Presentation	Cryoglobulin Detection			Rheumatological Factor (IU/mL) 0–20	C3 g/L 0.730–1.460	C4 g/L 0.100–0.400 L
					Isotype	Cryocrit (*n*, %)	Cryoglobulin Concentration, mg/L			
#1	Male 60 y	No	Skin	Chest tightness	Type II IgA κ	1.0	206.4	196.2	0.956	0.213
#2	Female 58 y	MGCS	Skin, kidney	Chest tightness	Type I IgG λ	2.0	1614.4	6.6	0.743	0.217
#3	Female 53 y	B-NHL	Skin, joint, lung	Chest tightness	Type I IgM κ	ND	3463.0	462.9	0.283	0.003
#4	Male 42 y	MGCS	Skin, peripheral nerves, kidney	Dyspnea, edema	Type I IgG κ	ND	ND	9.4	1.435	0.178
#5	Female 56 y	HCV	Skin, peripheral nerves, joint, lung, kidney	Dyspnea	Type II IgM κ	3.0	ND	463.9	0.489	0.002
#6	Male 26 y	HBV	Kidney	Orthopnea	Type II IgM κ	4.0	687.3	253.8	0.636	0.013
#7	Female 55 y	B-NHL	Skin, peripheral nerves, joint, kidney	Dyspnea, edema	Type II IgM κ	1.0	62.6	366.0	1.130	0.001
#8	Female 30 y	MGCS	Skin, peripheral nerves, joint, kidney	Chest tightness	Type I IgG λ	33.0	4576.7	3.0	1.056	0.145
#9	Male 35 y	HCV	Skin, peripheral nerves, GI	Dyspnea	Type II IgM κ	2.0	856.7	200.0	0.554	0.001
#10	Male 71 y	No	Kidney	Dyspnea	Type II IgM κ	<1.0	466.9	617.7	0.850	0.063
#11	Female 41 y	pSS	Kidney	Dyspnea, edema	Type II IgM κ	5.0	3535.1	410.8	0.777	0.048

B-NHL = B-cell non-Hodgkin lymphoma; HBV = hepatitis B virus; HCV = hepatitis C virus; GI = gastrointestinal tract; MGCS = monoclonal gammopathy of clinical significance; ND = not done; pSS = primary Sjögren’s syndrome.

**Table 2:** Baseline cardiac diagnostic investigations and findings in individual patients.

Patient	Electrocardiography	Echocardiography		CMR		Cardiac Biopsy
		LVEF	Others	LVEF	Others	
#1	Sinus tachycardia; low and flat T-waves	48%	Myocardial disease; LA enlargement	22.4%	LV and LA enlargement; diffuse reduction in LV motion; subepicardial LGE in LV lateral and inferior walls; pericardial effusion	ND
#2	Normal	62%	LV and LA enlargement; pericardial effusion	51.6%	Mild LV enlargement	ND
#3	Low and flat T-waves	48%	LV and LA enlargement; pericardial effusion	ND	ND	ND
#4	Normal	38%	Myocardial disease; LV and LA enlargement	38.8%	LV enlargement; diffuse reduction in LV motion	moderate perivascular mononuclear cell infiltration
#5	Sinus tachycardia; low and flat T-waves	60%	Pericardial effusion	56.5%	Mild septal thickening; pericardial effusion	ND
#6	Low and flat T-waves	37%	Myocardial disease; LV and LA enlargement; pericardial effusion; pulmonary hypertension	ND	ND	ND
#7	Normal	48%	Myocardial disease; LA enlargement; pulmonary hypertension	ND	ND	ND
#8	Normal	57%	Normal	ND	ND	ND
#9	Sinus tachycardia; T wave inversion in precordial leads	46%	Basal preserved, apical septal akinesia and global hypokinesis; LV and LA enlargement; pulmonary hypertension	ND	ND	ND
#10	Normal	59%	Myocardial disease; LA enlargement	ND	ND	ND
#11	Sinus tachycardia	49%	Myocardial disease; mild LV and LA enlargement	47.4%	Mid-myocardial LGE in LV free wall	ND

CMR = cardiac magnetic resonance; LA = left atrium; LGE = late gadolinium enhancement; LV = left ventricular; LVEF = left ventricular ejection fraction; ND = not done. LVEF was measured by M-mode echocardiography, with values <53% considered reduced. Pericardial effusion ≥ moderate was considered clinically significant; small or trace effusions were not classified as abnormal.

**Table 3:** Changes in cardiac biomarkers and echocardiographic parameters before and after treatment.

Patient	Before Treatment										After Treatment								
	^a^ cTnI, μg/L (0–0.056) ^b^ hscTnI ng/L (≤34)	NT-Pro-BNP (0–125 pg/mL)	BNP (0–100 ng/L)	LVEF	Chamber Dimensions				PASP mmHg	Pericardial Effusion	^a^ cTnI, μg/L (0–0.056) ^b^ hscTnI ng/L (≤34)	NT-Pro-BNP (0–125 pg/mL)	LVEF	Chamber Dimensions				PASP mmHg	Pericardial Effusion
					LVEDD	LVESD	LAD	RVD						LVEDD	LVESD	LAD	RVD		
#1	^a^ 0.051	>35,000	ND	48%	51	39	39	20	20	Trace	ND	ND	ND	ND	ND	ND	ND	ND	ND
#2	^a^ <0.017	209	ND	62%	56	37	46	24	20	Moderate	^a^ <0.017	24	58%	53	36	42	29	-	No
#3	^a^ <0.017	4332	190	48%	53	41	49	15	29	Moderate	ND	466	49%	52	39	40	23	-	Small
#4	^a^ <0.017	4526	442	38%	58	47	40	18	20	No	^a^ <0.017	58	64%	47	31	36	21	-	No
#5	^a^ 0.050	32,521	660	60%	47	32	37	23	20	Moderate	ND	1704	ND	ND	ND	ND	ND	ND	ND
#6	^a^ 0.023	32,826	ND	37%	59	47	44	28	45	Moderate	ND	6354	56%	57	40	35	23	20	No
#7	^a^ 0.120	>35,000	>5000	48%	53	40	40	20	45	Trace	^a^ 0.020	ND	63%	45	29	34	22	30	Trace
#8	^a^ 3.657	29,799	178	57%	50	35	34	19	20	No	^a^ <0.017	35	ND	ND	ND	ND	ND	ND	ND
#9	^a^ 0.454	5138	108	46%	55	42	42	29	71	No	^b^ 9.7	35	58%	43	30	34	19	20	No
#10	^a^ 0.020	20,391	340	59%	53	34	43	25	32	Small	^a^ <0.017	372	ND	ND	ND	ND	ND	ND	ND
#11	^b^ 54	>35,000	ND	49%	46	32	38	24	20	Small	^b^ 33	2237	67%	40	26	29	13	20	No

BNP = B-type natriuretic peptide; cTnI = cardiac troponin I; hscTnI = high-sensitivity cardiac troponin I; LAD = left atrial diameter; LVEDD = left ventricular end-diastolic diameter; LVEF = left ventricular ejection fraction; LVESD = left ventricular end-systolic diameter; ND = not done; NT-proBNP = N-terminal pro-B-type natriuretic peptide; RVD = right ventricular diameter. Pulmonary artery systolic pressure (PASP) was not routinely reported when <20 mmHg; such cases are denoted as “-” in the table.

**Table 4:** First-line treatment regimens and post-treatment outcomes in individual patients.

Patient	First-Line Treatment	Cardiac Response			Other Organ Response	Outcomes
		Cardiac Symptoms	Cardiac Biomarkers	Echocardiography		
#1	Declined treatment	NA	NA	NA	NA	Lost to follow-up
#2	BCD	Improved	Normal	LVEF 58%; LV and LA enlargement reduced	Improved	Cardiac and renal relapse, PFS 27 m
#3	RTX + CS	Improved	NT-proBNP decreased	LVEF 49%; LV and LA enlargement reduced; pericardial effusion decreased	Improved	Cardiac relapse, PFS 9 m, died of B-NHL
#4	BCD	Improved	Normal	LVEF 64%; normal echocardiography	Improved	Remission
#5	DRC + antiviral therapy for HCV infection	Improved	NT-proBNP decreased	ND	Improved	Remission
#6	CS + antiviral therapy for HBV infection	Improved	NT-proBNP decreased	LVEF 56%; myocardial disease improved; LV enlargement reduced; PASP normalized	Improved	Cardiac and renal relapse due to HBV reactivation, PFS 42 m
#7	DRC	Improved	cTnI decreased to normal	LVEF 63%; myocardial disease resolved; LA enlargement reduced; PASP decreased	Improved	Cardiac, renal and peripheral nerve relapse, PFS 30 m
#8	PE + BCD	Improved	Normal	ND	Improved	Remission
#9	R-CVP+ antiviral therapy for HCV infection	Improved	Normal	LVEF 58%; wall motion normalized; LV and LA enlargement resolved; PASP normalized	Improved	Remission
#10	DRC	Improved	Normal	ND	Improved	Remission
#11	RTX + CS	Improved	NT-proBNP decreased	LVEF 67%; normal echocardiography	Improved	Relapse, new skin involvement, PFS 30 m

Cardiac biomarkers include N-terminal pro-B-type natriuretic peptide (NT-proBNP) and cardiac troponin I (cTnI). BCD = bortezomib, cyclophosphamide, and dexamethasone; B-NHL = B-cell non-Hodgkin lymphoma; CS = corticosteroid; DRC = dexamethasone, rituximab, and cyclophosphamide; HBV = hepatitis B virus; HCV = hepatitis C virus; LA = left atrium; LV = left ventricular; LVEF = left ventricular ejection fraction; NA = not applicable; ND = not done; PASP = pulmonary artery systolic pressure; PE = plasma exchange; PFS = progression-free survival; RTX = rituximab; R-CVP = rituximab, cyclophosphamide, vincristine, and prednisone.

**Table 5:** Second-line treatment regimens and outcomes in patients who relapsed.

Patient	Second-Line Treatment	Cardiac Response			Other Organ Involvement	Outcome
		Cardiac Symptoms	Cardiac Biomarkers	Echocardiography		
#2	Rd	Improved	Normal	ND	Improved	Remission
#6	CS + antiviral therapy for HBV infection	Improved	Normal	ND	Improved	Remission
#7	DRC	Worsened	Worsened	ND	Worsened	Died of respiratory failure
#11	RTX	Stable	Stable	LVEF 65%; normal echocardiography	Improved	Remission

CS = corticosteroid; DRC = dexamethasone, rituximab, and cyclophosphamide; HBV = hepatitis B virus; LVEF = left ventricular ejection fraction; ND = not done; Rd = lenalidomide plus dexamethasone; RTX = rituximab.

## Data Availability

The original contributions presented in this study are included in the article. Further inquiries can be directed to the corresponding author.

## Supplementary Materials

The following supporting information can be downloaded at: https://www.mdpi.com/article/10.3390/jcm15135262/s1. Figure S1. Patient #1: Cine and late gadolinium enhancement (LGE) images (A); subepicardial LGE in the left ventricular lateral and inferior walls (B,C). Patient #11: Cine and LGE images (D); mid-myocardial LGE in the left ventricle free wall (E,F).
