# Supplementary material for: Cardiac Involvement in Cryoglobulinemia: Clinical Characteristics, Radiological Features, and Outcomes
Source: J Clin Med. 2026 Jul 6;15(13):5262. doi: 10.3390/jcm15135262 (PMC13362785; doi:10.3390/jcm15135262)
Supplement: Supplementary file 1 [file jcm-15-05262-s001.zip › jcm-4367663-supplementary.pdf]

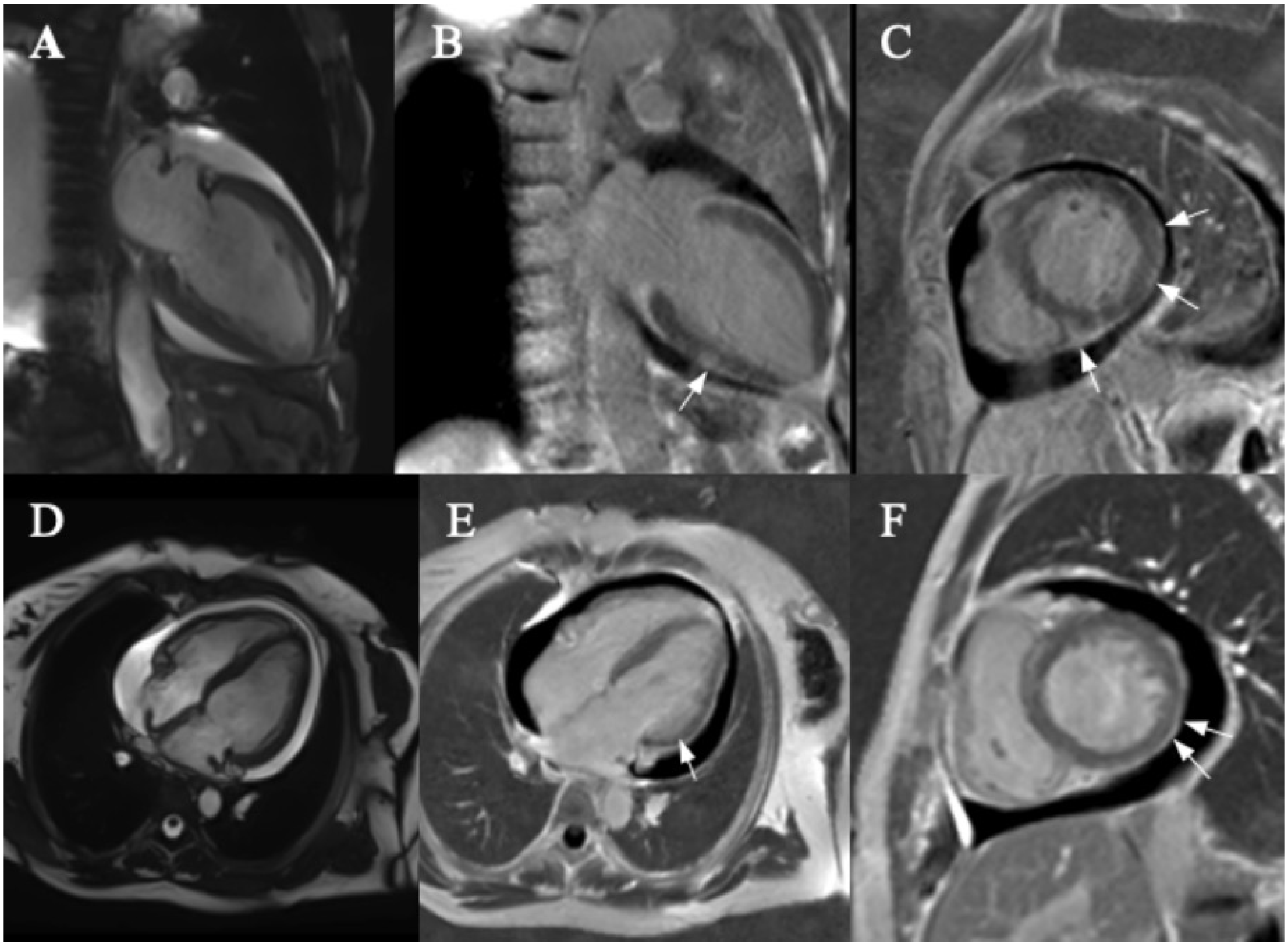

Figure S1. **Patient #1**: Cine and late gadolinium enhancement (LGE) images (**A**); subepicardial LGE in the left ventricular lateral and inferior walls (**B**,**C**). **Patient #11:** Cine and LGE images (**D**); mid-myocardial LGE in the left ventricle free wall (**E**,**F**).
